# The Association of OASL and Type I Interferons in the Pathogenesis and Survival of Intracellular Replicating Bacterial Species

**DOI:** 10.3389/fcimb.2017.00196

**Published:** 2017-05-19

**Authors:** Gina Leisching, Ian Wiid, Bienyameen Baker

**Affiliations:** SAMRC Centre for TB Research, DST/NRF Centre of Excellence for Biomedical Tuberculosis Research, Division of Molecular Biology and Human Genetics, Faculty of Medicine and Health Sciences, Stellenbosch UniversityCape Town, South Africa

**Keywords:** OASL, OAS, cGAS, STING, Type I IFN, mycobacteria, pathogenesis

## Abstract

The type I IFN response quickly became associated with its role in the innate immune response to viral infection. The past few years have seen the significance of IFNs expand in breadth to include non-viral pathogens. Previous work has identified that following viral infection, type I IFN signaling induces the production of the 2′-5′-oligoadenylate synthetase (OAS) family, which include OAS1, OAS2, OAS3, and OAS-like (OASL) protein. OASL was identified to be strongly induced following viral infection through engaging the RNA sensor RIG-I and increasing signaling through this pathway to enhance the anti-viral type I IFN response. Surprisingly, infection with viral dsDNA revealed an IFN inhibitory role and therefore pro-viral function of OASL through the inhibition of the cGAS cytosolic DNA sensing mechanism. Intracellular bacteria are able to activate the cytosolic DNA sensing pathway, however the role of OASL during bacterial infection is largely unknown. Vacuolar pathogenic microbes such as mycobacteria induce *OASL* early post infection, where it functions in a prosurvival fashion by inhibiting autophagic mechanisms and antimicrobial peptide expression. This suggests an underestimated role of OASL in the innate immune response to infection with a variety of pathogens and points to OASL-associated modulation of the type I IFN response. OASL may therefore play a critical role in defining the outcome of infection. We provide a brief update on the recent developments of the OAS family of proteins in response to DNA and RNA virus infections, as well as discuss evidence of *Oasl* expression in response to a number of cytosolic and vacuolar replicating bacterial pathogens.

## Introduction

The 2′-5′-oligoadenylate synthetases (OAS) were the first interferon (IFN)-induced antiviral proteins described that are regulated by both type I and II IFNs (Zhou et al., 1997; Eskildsen et al., 2003). Originally, type I IFNs were discovered as mediators of the host response to viral infection, however it is now well-know that type I IFN induction is central to the genetic response of both viral and bacterial pathogens (Uematsu and Akira, 2006). The OAS family of proteins consists of OAS1, OAS2, OAS3, and OAS-like (OASL) protein. The OAS proteins exhibit anti-viral functions through indirectly impeding the translation of viral nucleic acids through the activation of RNase L. OASL on the other hand, is related to the OAS family by a N-terminal OAS-like domain but lacks synthetase activity. The lack of enzymatic activity is by no means an indication of a lack of function, as recently an anti-viral role was ascribed that is underpinned by the ability of OASL to enhance of IFN I signaling (Melchjorsen et al., 2009; Zhu et al., 2014, 2015; Choi et al., 2015). On the heels of this discovery, it was observed that this antiviral function is specific to infection with RNA viruses, however when challenged with a DNA virus, OASL exerts a prosurvival function through the inhibition of IFN I signaling (Ghosh et al., 2016). Not only does this reveal a contrasting bifunctional role of OASL, it also suggests that it is able to modulate the type I IFN response when challenged with RNA and DNA pathogens. Further, this suggests the possibility that intracellular pathogens such as bacteria, once phagocytosed, induce a similar, if not an identical response when bacterial DNA enters the host cytosol. The biological role of the OAS family during microbial infection is not well documented, however recent work has revealed a dichotomous function of OASL in promoting intracellular mycobacterial survival that is closely related with stimulator of interferon genes (STING; also known as TMEM173), IFN secretion, the RNase L endocytic pathway, and autophagy. Here we provide a brief update on the recent developments of the OAS family of proteins in response to DNA and RNA virus infections, as well as discuss evidence of *Oasl* expression in response to a number of intracellular bacterial pathogens.

## IFN/RNase L/OASL antiviral mechanism

**Figure 1:**
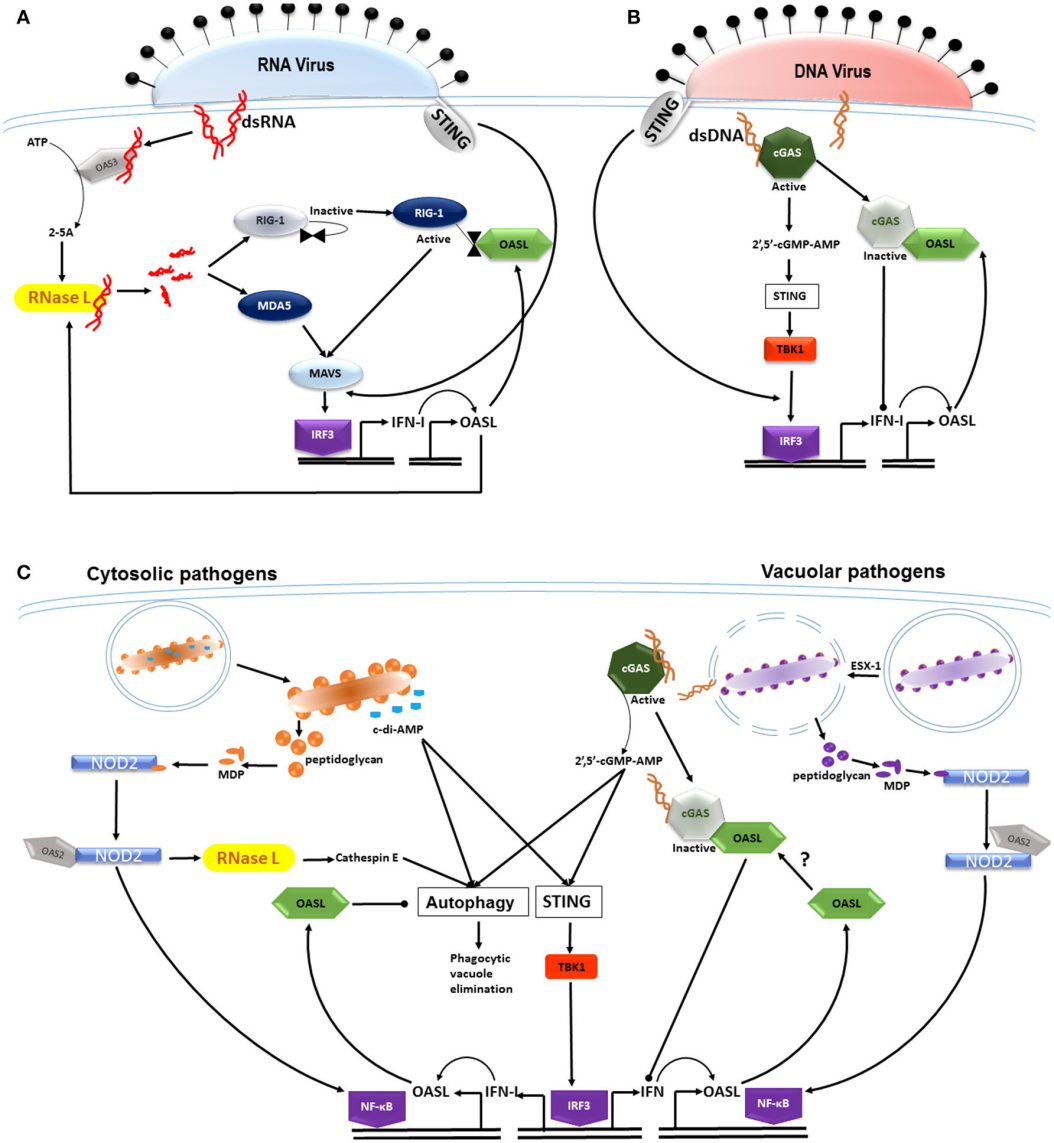
**OASL differentially modulates type I IFN expression in response to RNA and DNA in the host cytosol. (A)** Viral dsRNA binds to and activates OAS3 producing 2–5A activators of RNase L. RNase L functions to cleave viral RNA which then activate the cytoplasmic recognition receptors RIG-1 and MDA5. Subsequent signaling through the MAVS adaptor induces the translocation of interferon regulatory factor (IRF) 3 to the nucleus, resulting in the transcription of type I IFNs (STING-independent). IFN signaling induces OASL production, which when present in the cytosol, binds to and activates RIG-1 signaling, thereby enhancing IFN secretion and controlling viral replication. **(B)** The entry of viral dsDNA is sensed by the cytoplasmic DNA sensor cGAS, which through cGAMP induces the translocation of IRF-3 to the nucleus in a STING-independent manner. IFN induction and signaling induces OASL, which then binds to and inactivates cGAS, thereby inhibiting IFN production, allowing for the infection to persist. Although, IFN-I induction induced by both RNA and DNA viruses is STING-independent, STING is able to sense membrane fusion events at the cell membrane where it is able to induce IRF3. **(C)** Cyclic dinucleotides in the form of c-di-AMP are produced by diverse bacterial species and activate autophagic mechanisms as well as the STING/TBK1/IRF3 axis in a CpG-independent manner. The innate response to both cytosolic and vacuolar bacterial pathogens is enhanced through muramyl dipeptide (MDP), a breakdown product of bacterial peptidoglycan. MDP activates NOD2 which is then bound by OAS2, subsequently activating the inflammasome through NF-κB. NOD2 enhances RNase L activity which increases expression of cathepsin E- a mediator in the phagocytosis of bacteria. Vacuolar pathogens such as *M. tuberculosis* are able to perforate the phagosome, a process mediated by the ESX-1 secretion system. Bacterial dsDNA then gains entry into the cytosol binding to and activating cGAS, thus inducing signaling through the STING/TBK1/IRF3 axis. OASL production follows IFN signaling which inhibits autophagic mechanisms and antimicrobial peptide expression, thus promoting intracellular bacterial survival. It is unknown whether there is an association between cGAS and OASL and whether this influences type 1 IFN secretion.

The control of viral infection is essentially modulated in two parts, which upon entry of viral dsRNA, become activated and positively stimulate one another (Figure 1A). The first is the canonical OAS3/RNase L pathway which relies on the binding of OAS3 directly to dsRNA (Li et al., 2016). Previously it was thought that OAS1, 2, and 3 were involved in activating RNase L during viral infection, until recently Li and colleagues observed that OAS3 is mainly responsible for producing the 2–5A RNase L activators (Li et al., 2016). The control of infection is then achieved through 2–5A binding to RNase L, causing its dimerization and enabling its endoribonuclease activity (Tanaka et al., 2004). The second antiviral mechanism is stimulated by these cleavage products which then activate the cytoplasmic recognition receptors known as the retinoic acid-inducible gene I (RIG-1) receptor (RLR) (Yoneyama et al., 2004) and the melanoma differentiation-associated gene 5 (MDA5). The downstream signaling adaptor MAVS induces the translocation of interferon regulatory factor (IRF) 3 to the nucleus and induces the transcription of type I IFNs. The production of type I IFNs then enhances the activation of the RNase L degradative pathway (Silverman, 2007) and controls viral replication. It was observed that type I IFN signaling induces *OASL* through IRF3. OASL, which when present in the cytosol, binds directly to RIG-1 and mimics polyubiquitin, thereby sensitizing RIG-1 activation by viral RNA and enhancing antiviral signaling (Zhu et al., 2014). Thus during infection, OASL functions to enhance type I IFN signaling and suppress replication of RNA viruses. It has since been put forward that the anti-viral capability of OASL be harnessed with the potential for developing broad acting antiviral therapy (Zhu et al., 2015).

## cGAS/IFN/OASL proviral mechanism

Unlike infection with RNA viruses, much less is known about the host cells response to infection with DNA viruses. Cyclic GMP-AMP (cGAMP) synthase (cGAS) is a sensor of cytoplasmic DNA, usually of microbial origin and has recently been thoroughly reviewed (Chen et al., 2016; Patrick et al., 2016). Of interest however is the structurally homologous nature of cGAS to the OASs and the recent observation that it is able to bind directly to viral DNA(Sun et al., 2013). Once bound, 2′,5′-cGMP-AMP (cGAMP) is produced and signals through TBK1 and STING, inducing type I IFN production through the activation of IRF3 (Figure 1B). As with RNA virus infections, the presence of IFN signaling then rapidly induces *OASL* which binds to, and deactivates cGAS (Ghosh et al., 2016). The deactivation of cGAS consequently inhibits IFN production which then allows for the persistence of the DNA virus infection. It is accepted that signaling through cGAS during the initial stages of infection is accompanied by the downstream activation of STING where it plays an essential role in type I interferon-dependant innate immunity in response to intracellular DNA (Ishikawa et al., 2009). However, it is suspected that cGAS-dependant IFN induction by DNA viruses such as Vaccinia and HSV is independent of STING stimulation but this is yet to be confirmed (Ghosh et al., 2016). It is however important to note that STING is able to sense membrane-fusion events that are associated with viral entry into the host cell that is independent of nucleic acid sensing, but is still able to induce type I IFNs (Holm et al., 2012). Moreover, STING is unlikely to be required for IFN induction in response to RNA or RNA viruses presumably because viruses can activate IRF3 through RIG-1 and its downstream signaling adaptor MAVS (Chiu et al., 2009). This points to the possibility of a virus-specific response whereby STING-dependent type 1 IFN production is bypassed.

## cGAS/STING/IFN/OASL probacterial mechanism

Phagocytic cells are not only able to discriminate between viral and bacterial infection, but also extracellular microbes from intracellular replicating pathogens. Pattern recognition receptors (PRRs) are central to distinguishing these signals and sensing conserved motifs presented by microbes (Gordon, 2002; Meylan et al., 2006; Kawai and Akira, 2010). The membrane-bound Toll-like receptors (TLRs) monitor extracellular activity and phagolysosomal compartments by recognizing pathogen associated molecular patterns (PAMPs) that include flagella, bacterial lipoprotein, lipopolysaccharide (LPS), and CpG DNA (Akira et al., 2006) and are responsible for inducing proinflammatory genes. The soluble, cytosolic NOD-like receptors (NLRs) recognize cell wall fragments from both Gram-negative and Gram-positive bacteria (Hasegawa et al., 2006; Shaw et al., 2008), as well as acid-fast mycobacteria (Jo, 2008) that activate the inflammasome (Figure 1C). Muramyl dipeptide (MDP), a peptidoglycan break down product, is one such cell wall fragment which binds to and activates NOD2 (Girardin et al., 2003). It was observed that OAS2 binds NOD2 and enhances RNase L activity (Dugan et al., 2009) which increases expression of cathepsin E, an important mediator of autophagy following the phagocytosis of bacteria (Tsukuba et al., 2013). Additionally, the activation of NOD2 leads to the activation of the NF-κB transcription factor which is associated with the release of cytokines and control of the infection. Once phagocytosed, most pathogenic intracellular bacteria are able to mediate the phagosome breach, which in the process leads to content leakage in the form of dsDNA into the host cytosol(Vance et al., 2009; Manzanillo et al., 2012). Vacuolar pathogens such as *Mycobacterium tuberculosis* perforate the phagosome, a process mediated by the ESX-1 secretion system(Manzanillo et al., 2012). In this way, extracellular mycobacterial DNA is able to enter the host cytosol, inducing IFN-β signaling through the *Sting/Tbk1/Irf3* axis. Bacterial pathogens such as *Listeria monocytogenes* that replicate in the cytoplasm induce IFN-β transcription as part of the cytosolic surveillance pathway (CSP) (Henry and Monack, 2007) which induces signaling through the very same axis. Thus the inflammatory response induced by infection with cytosolic or vacuolar pathogens converge through this signaling axis. The signaling events downstream of this have however remained unclear until recently.

As with viral dsDNA, it was observed that the entry of foreign bacterial dsDNA is met with the DNA sensor cGAS, which then signals through cGAMP, activating the *Sting/Tbk1/Irf3* axis which will culminate in type I IFN secretion. It is now known that IFN-α IFN-β secretion in response to bacterial infection is in fact a pathogenic mechanism that enhances bacterial replication (Stanley et al., 2007). Moreover, OASL is also produced as a result of signaling through this axis and aids in promoting a favorable intracellular milieu for bacterial survival. In line with this, the biological role of OASL during mycobacterial infection, specifically with the vacuolar pathogen *M. leprae* was recently elucidated (de Toledo-Pinto et al., 2016). The authors suggest that the presence of OASL is required for intracellular *M. leprae* survival which appears to be dependent on inhibiting autophagic mechanisms, thus preventing clearance of the microbe. Since this is the first study observing the OASL-(vacuolar) microbe link, the next question that arises is whether OASL is relevant during infection with other vacuolar pathogens and those which replicate in the cytoplasm. A search of the literature revealed the upregulation of *OASL* or the murine equivalent *Oasl2* through transcriptome analysis (Table 1) in a number of infection models. Its upregulation has been observed in response to a number of Gram-positive and Gram-negative bacteria from as early as 4 hpi. These pathogens include *Chlamydia trachomatis* (Lad et al., 2005), *Chlamydia pneumonia* (Eickhoff et al., 2007), *L. monocytogenes* (Kutsch et al., 2008), *Brucella abortus* (Roux et al., 2007), and *Lactobacillus acidophilus* (Weiss et al., 2010). Although, this search was not exhaustive, it suggests that OASL more than likely plays a role during infection with a wide range of bacterial species. What needs to be determined is whether OASL binds to and inactivates cGAS, thus inhibiting STING-dependant type I IFN production. This is what is observed during infection with HSV and Vaccinia DNA viruses (Ghosh et al., 2016), however an identical response cannot be assumed since it was determined that *OASL* knock-down did not influence IFN-β release during *M. leprae* infection (de Toledo-Pinto et al., 2016).

**Table 1:** **Vacuolar and cytosolic bacterial pathogens that induce ***OASL/Oasl2*****.

**Stimuli**	**Host**	**Hours P.I**	**References**	**Analysis platform**	**Gene**
*Chlamydia trachomatis*	HeLa 229	16	Lad et al., 2005	Microarray	*OASL*
*Chlamydia pneumoniae*	HeLa	24	Eickhoff et al., 2007	Microarray	*OASL*
*Listeria monocytogenes*	C57BL/6 mice	30	Kutsch et al., 2008	Microarray	*Oasl2*
*Brucella abortus*	C57BL/6 mice	36	Roux et al., 2007	Microarray	*Oasl2*
*Lactobacillus acidophilus*	Murine DC	4	Weiss et al., 2010	Microarray	*Oasl2*
*Mycobacterium leprae*	Primary Schwann cells	48	de Toledo-Pinto et al., 2016	Microarray	*OASL*
*Mycobacterium tuberculosis*	Murine BMDM	12	Leisching et al., 2016	RNAseq	*Oasl2*

The balance between type I and type II IFNs is particularly important for controlling intracellular bacterial infections, notably *M. tuberculosis, M. leprae*, and *L. monocytogenes*, and since OASL plays a significant role in modulating type I IFN production is can be said that the expression of this protein may be central to controlling viral and bacterial infections. Collectively, the above evidence clearly points to an underestimated role of OASL in the innate immune response to infection with a variety of pathogens. Not only is OASL expression closely related to the secretion of type I IFNs, its role is clearly diverse with it interacting closely with the RIG-I pathway, autophagy, and DNA sensing through cGAS.

## Author contributions

GL wrote the manuscript. IW and BB provided substantial contributions to the framework of the manuscript, as well as revision of the work, and approval of the final draft.

### Conflict of interest statement

The authors declare that the research was conducted in the absence of any commercial or financial relationships that could be construed as a potential conflict of interest.
